# Prevalence of vitamin D status and its association with overweight or obesity in a population of Colombian children and adolescents

**DOI:** 10.1017/jns.2020.47

**Published:** 2020-11-26

**Authors:** Lyda Z. Rojas, Doris C. Quintero-Lesmes, Edna M. Gamboa-Delgado, Elizabeth Guio, Norma C. Serrano

**Affiliations:** 1 Fundación Cardiovascular de Colombia, Dirección de Investigaciones, Calle 155A No. 23–58, Urbanización el Bosque, Floridablanca, Colombia; 2 Universidad Industrial de Santander, Escuela de Nutrición y Dietética, carrera 32 No. 29–32, Bucaramanga, Santander, Colombia

**Keywords:** Vitamin D, 25-OH-D, Adolescents, Children, Cases and Control Study

## Abstract

The present study aimed to estimate the prevalence of 25-OH-D status (insufficiency and deficiency) in children and adolescents residing in Bucaramanga, Colombia and to determine its association with excess weight. A case–control study was nested in the SIMBA II cohort in children and adolescents between the ages of 11 and 20 years old. Cases were defined as those children and adolescents with overweight or obesity. The control group was composed of children and adolescents from the same population sample with similar sociodemographic and economic characteristics but without overweight or obesity diagnosis. 25-hydroxyvitamin D (25-OH-D) was quantified in serum using a chemiluminescent microparticle immunoassay. Logistic regression models were used to assess the association between vitamin D status and overweight or obesity adjusted for the main confounding variables. A total of 494 children and adolescents cases were 138 (52⋅17% boys and 47⋅83% girls; median age 16⋅0 [Q1 15; Q3 18]). The median BMI S-Score minors age in the cases was 1⋅36 [Q1 1⋅06; Q3 2⋅00] and BMI (kg/m^2^) 28⋅0 [Q1 26⋅2; Q3 30⋅8]. The prevalence of vitamin D in the cases was deficiency 16⋅67%, insufficiency 57⋅25%, sufficiency 26⋅09. 25-OH-D insufficiency was associated with overweight or obesity after adjusting for the main confounding variables (OR 1⋅73; 95% CI 1⋅05–2⋅84). Our study concludes that the 25-OH-D insufficiency is common in children and adolescents in Bucaramanga, Colombia, and it was associated with overweight or obesity.

## Introduction

Vitamin D [25-hydroxyvitamin D (25-OH-D)] is essential for the normal development and metabolism of bone tissue, as well as for calcium and phosphorous homeostasis^(1,2)^. Similarly, 25-OH-D has several implications for the immune, nervous, cardiovascular and endocrine systems adequate functioning^(2)^. The 25-OH-D deficiency has a high prevalence in children and adolescents worldwide and it has been related to the risk of developing conditions, such as rickets, osteomalacia, asthma, autism, cancer, depression, obesity and metabolic syndrome, among others^(3–5)^. However, these associations have mainly been observed in observational studies, which are at high risk of confounding from other healthy behaviours as a decrease in sun exposure (due to a decrease in the 25-OH-D synthesis of the skin) and an insufficient intake of foods rich in 25-OH-D^(6,7)^.

Specifically, the evidence in the literature has been inconsistent regarding the relationship between 25-OH-D and obesity. Observational studies have reported lower levels of 25-OH-D in obese subjects compared to those with normal weight; however, exists published evidence proposing an opposite correlation between 25-OH-D and body fat, while other studies have found no association^(8)^. Recently, one study performed a bidirectional Mendelian randomisation analysis. An approach that limits confounding factors to evaluate the causal relationship between obesity and vitamin D status. Its results suggested that a higher body mass index (BMI) leads to lower levels of 25-OH-D, while that any reduction in 25-OH-D levels is likely to have a small or minor effect on the risk of increasing BMI^(9)^. Likewise, a recent meta-analysis showed that 25-OH-D levels are inversely correlated with the percentage of body fat, but the supplementation with cholecalciferol had no significant effect on the same outcome^(8)^. In contrast, a recently published clinical trial concluded that decreasing 25-OH-D deficiency in overweight and obese children by offering supplemental vitamin D3 (1000 or 2000 IU/day) led to significant reductions in blood pressure and glucose levels, and an improvement in insulin sensitivity, when compared to a 600 IU/day dosage. The previously mentioned suggests that the optimisation of vitamin D status could improve cardiovascular health in this population^(10)^.

In Colombia, a high prevalence of 25-OH-D deficiency has been documented^(11)^. According to a study carried out in Bogotá, Colombia, the prevalence of 25-OH-D deficiency in children aged 5–12 years was 10⋅2 %, while 25-OH-D insufficiency was observed in 46⋅4 % of the total^(12)^. Additionally, a recent systematic review on the global status of vitamin D revealed the lack of data in infants, children and adolescents worldwide, but mainly in South American and African countries^(3)^. Consequently, the objective of the present study was to estimate the prevalence of 25-OH-D status (insufficiency and deficiency) in children and adolescents residing in Bucaramanga, Colombia and to determine its association with excess weight. The present results will serve as a starting point for conducting a pilot clinical trial where children and adolescents will be supplemented with vitamin D to elucidate the possible causal effect of 25-OH-D status on excess weight in our population.

## Methods

### Study design and population

**Fig. 1.**
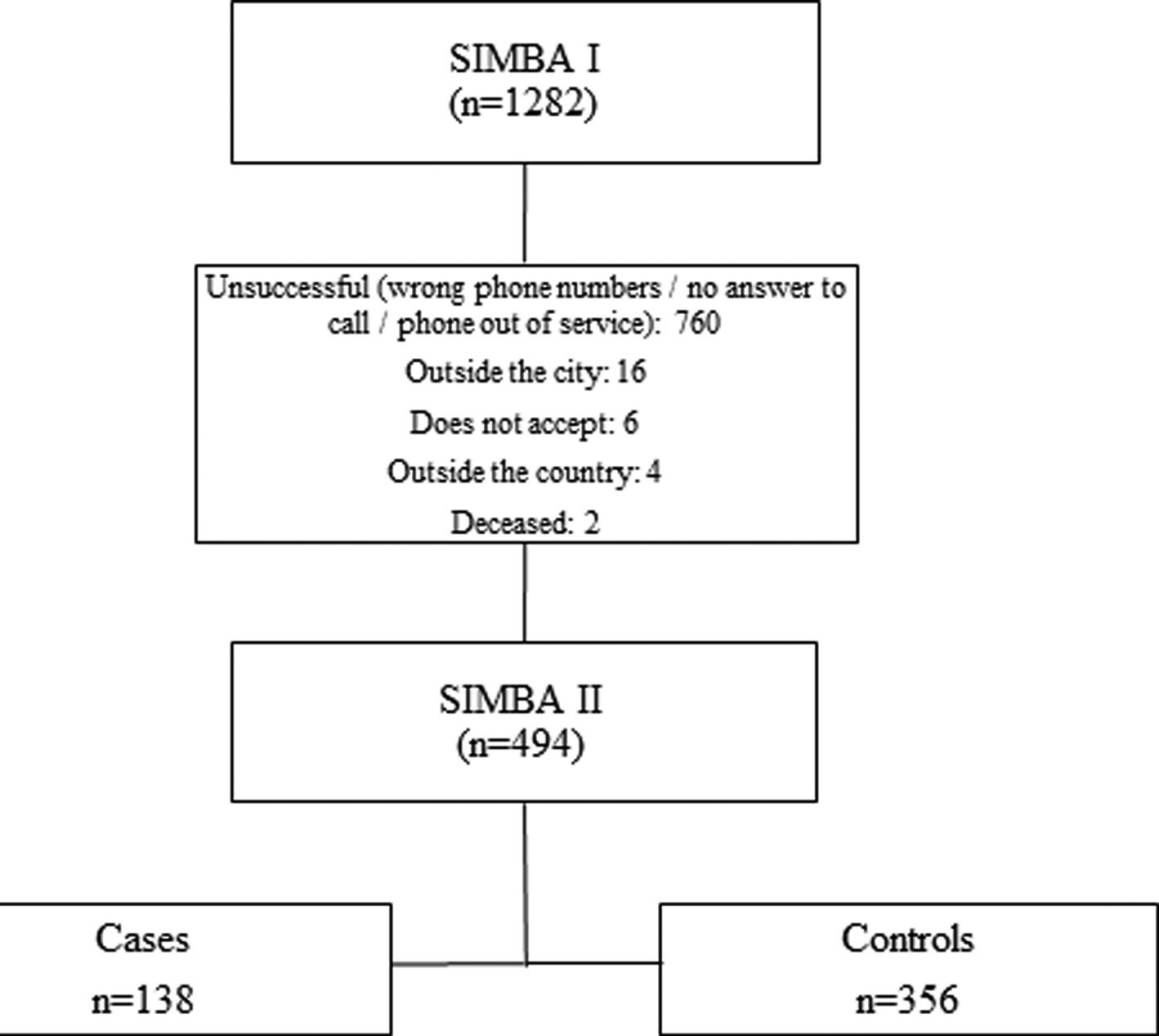
Sample selection flowchart.

This case–control study was nested in the SIMBA cohort, which started in 2006 enrolling a total of 1282 children between 6–10 years old in the city of Bucaramanga, Colombia^(13)^. The SIMBA II project was executed between 2013 and 2017. This second cohort aimed to follow up 494 children and adolescents between the ages of 11 and 19 years old(^14^). All SIMBA II participants were involved in this analysis (Fig. 1).

This study was conducted according to the guidelines laid down in the Declaration of Helsinki and all procedures involving human subjects/patients were evaluated and approved by the Research Ethics Committee of the Fundación Cardiovascular de Colombia, with the number register 396 of 9 April 2016. The parents or legal representatives of the children gave their informed written consent. Additionally, all school-age children gave their assent and signed an agreement.

### Definition of cases and controls

Cases were defined as those children and adolescents with overweight or obesity. For adolescents between 18 and 20 years of age, overweight or obesity was defined as the BMI (ratio between weight in kilograms and height in square metres [kg/m^2^]), defining those whose result was ≥25 kg/m^2^ as having overweight or obesity. In participants of <18 years old, BMI was established by calculating the *Z* scores standardised by age and sex, which were defined using the growth standards of the World Health Organization (WHO), as the reference population. The cut-off points to classify nutritional status according to the BMI *Z* scores were normal BMI ≥−2⋅00 and ≤0⋅99 and excess weight >0⋅99(^15^). The control group was composed of children and adolescents from the same population sample with similar sociodemographic and economic characteristics but without overweight or obesity diagnosis.

### Sample collection and quantification of 25(OH)D: main exposure

Blood specimens were drawn from the antecubital vein using a vacutainer tube (Becton-Dickinson) following the venipuncture procedure. Blood components were separated by centrifugation within the next hour of collection, and serum samples were stored at −80°C until 25(OH)D quantification. Participant's serum samples were assayed for a total of 25(OH)D. 25-hydroxyvitamin D (25-OH-D) was quantified in serum using a chemiluminescent microparticle immunoassay (CMIA) (ARCHITECT 25-OH Vitamin D – Abbott). The laboratory is recognised and certified Randox International Quality Assessment Scheme (RIQAS). The following cut-off points suggested by Holick *et al.* were used: deficiency for values below 20 ng/ml (<50 nmol/l), insufficiency from 21 to 29 ng/ml (51–74 nmol/l) and sufficiency for values over 30 ng/ml (≥75 nmol/l)^(16)^.

### Additional variables

Information related to sociodemographic, nutritional and anthropometric characteristics and physical activity was also evaluated. Besides, blood samples were obtained to measure fasting blood glucose, lipid profile, uric acid and insulin levels. The method of the measurement of these variables has been previously described^(13)^.

### Statistical analysis

The prevalence of vitamin D status and overweight or obesity was calculated with the respective 95 % confidence intervals (CI). Categorical sociodemographic and clinical characteristics in each group were compared using chi-square or chi-trend tests, while the Mann–Whitney *U* test was used for comparing continuous variables. Logistic regression models were used to assess the association between vitamin D status and overweight or obesity adjusted for the main confounding variables. The Hosmer–Lemeshow goodness of fit test was used to evaluate the models. Values of *P* < 0⋅05 were considered statistically significant. All analyses were performed in Stata v15 statistical software.

## Results

A total of 494 children and adolescents (age range 11–20 years) were involved in this analysis. Cases and controls were similar in terms of sociodemographic and behavioural characteristics related to lifestyle, except for socioeconomic status (Table 1). 25-OH-D levels ranged from 13⋅2 to 66⋅2 ng/ml (median 27⋅2; Q1 22⋅2–Q3 32⋅7 ng/ml) and were lower in patients with overweight when compared to those with a normal body weight (median 25⋅9; Q1 21⋅8; Q3 30⋅2 ng/ml and median 27⋅6; Q1 22⋅3; Q3 33⋅2 ng/ml, *P*=0⋅033, respectively). Sociodemographic and clinical characteristics were compared between cases and controls in Table 1.

**Table 1.** Sociodemographic and clinical characteristics of cases and controls (*n* 494)

Variable	Cases, *n* 138	Controls, *n* 356	*P*-value
Sex, *n* (%)			
Males	72 (52⋅17)	166 (46⋅89)	0⋅292
Females	66 (47⋅83)	188 (53⋅11)	
Age (years), median [Q1–Q3]	16 [15–18]	16 [15–18]	0⋅580
Socioeconomic status, *n* (%)			
Low	67 (48⋅55)	229 (64⋅33)	0⋅001
Medium	71 (51⋅45)	127 (35⋅67)	
Alcohol intake (Yes), *n* (%)	77 (58⋅33)	210 (60⋅00)	0⋅740
Diet rich in 25-OH-D (Yes), *n* (%)	102 (75⋅00)	258 (72⋅68)	0⋅602
BMI S-Score, median [Q1–Q3]	1⋅36 [1⋅06; 2⋅00]	−0⋅04 [−0⋅78; 0⋅48]	0⋅000
BMI (kg/m^2^), median [Q1–Q3]	28⋅0 [26⋅2–30⋅8]	21⋅1 [19⋅2–23⋅3]	0⋅000
Waist-to-hip ratio, median [Q1–Q3]	0⋅84 [0⋅78–0⋅89]	0⋅83 [0⋅77–0⋅88]	0⋅118
Vigorous physical activity (For at least 20 min), *n* (%)			
Never	35 (26⋅12)	78 (22⋅74)	0⋅841
1–3 days/week	64 (47⋅76)	182 (53⋅06)	
≥4 days/week	35 (26⋅12)	83 (24⋅20)	
Television viewing time, *n* (%)			
≤1 h/day	46 (37⋅70)	124 (37⋅35)	0⋅680
2–3 h/day	59 (48⋅36)	151 (45⋅48)	
≥4 h/day	17 (13⋅93)	57 (17⋅17)	
Computer use time, *n* (%)			
≤1 h/day	23 (17⋅83)	49 (14⋅54)	0⋅619
2–3 h/day	34 (26⋅36)	95 (28⋅19)	
≥4 h/day	72 (55⋅81)	193 (57⋅27)	
SBP (mmHg), median [Q1–Q3]	109 [102–119]	107 [101–115]	0⋅026
DBP (mmHg), median [Q1–Q3]	64 [59–70]	64 [58–69]	0⋅237
Glycemia (mg/dl), median [Q1–Q3]	91 [87–95]	91 [87–95]	0⋅520
Insulin levels (μU/ml), median [Q1–Q3]	12 [9–18]	9 [6–13]	0⋅000
HOMA-IR (UI/ml), median [Q1–Q3]	2⋅5 [1⋅9–4⋅0]	2⋅0 [1⋅5–2⋅9]	0⋅000
Total-C (mg/dl), median [Q1–Q3]	157 [142–177]	156 [140–175]	0⋅675
LDL (mg/dl), median [Q1–Q3]	94 [77–112]	90 [76–107]	0⋅220
HDL (mg/dl), median [Q1–Q3]	45 [39–52]	48 [41–56]	0⋅001
Triacylglycerols (mg/dl), median [Q1–Q3]	88 [61–123]	78 [59–103]	0⋅041
25-OH-D levels (ng/ml), median [Q1–Q3]	25⋅9 [21⋅8–30⋅2]	27⋅6 [22⋅3–33⋅2]	0⋅033
25-OH-D status, *n* (%)			
Deficiency (≤20 ng/ml)	23 (16⋅67)	59 (16⋅57)	0⋅008
Insufficiency (21–29 ng/ml)	79 (57⋅25)	161 (45⋅22)	
Sufficiency (≥30 ng/ml)	36 (26⋅09)	136 (38⋅20)	

Q1–Q3: first and third quartile.

**Fig. 2.**
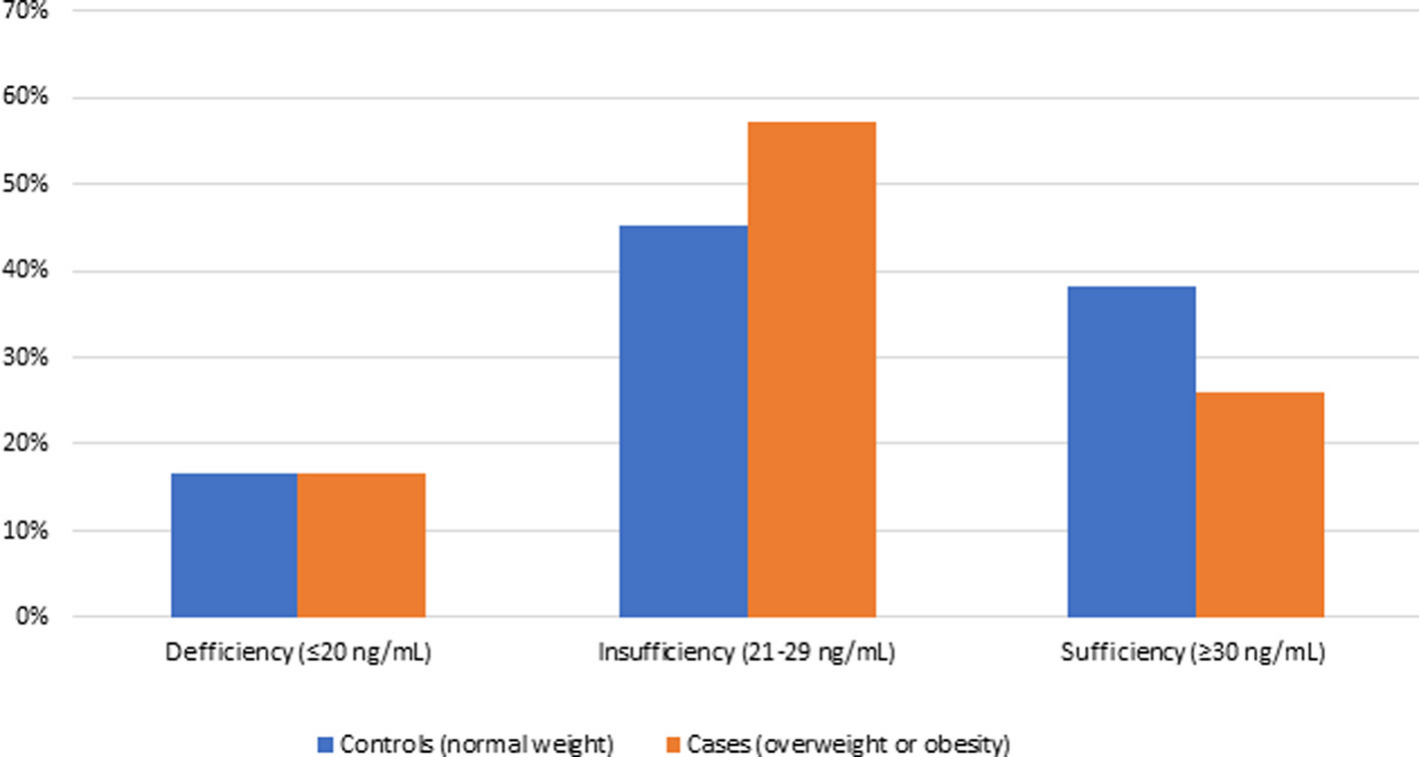
Prevalence of vitamin D status in children and adolescents with normal body weight *v.* cases with overweight or obesity.

The overall prevalence of overweight or obesity was 27⋅9 % (95 % CI 24⋅0–32⋅1), while the prevalence of 25-OH-D deficiency/insufficiency status was 65⋅2 % (95 % CI 59⋅7–70⋅4). Moreover, the prevalence of 25-OH-D deficiency was similar in both cases and controls (16⋅6 *v*. 16⋅5 %, respectively. *P*=0⋅980), while insufficiency was more frequent in overweight participants (57⋅2 *v*. 45⋅2 %, respectively, *P*=0⋅016) and sufficiency was more common in individuals with normal weight (38⋅2 *v*. 26⋅1 %, respectively, *P*=0⋅011) (Table 2 and Fig. 2).

**Table 2.** Prevalence of vitamin D status in each study group (*n* 494)

Vitamin D	All *n* 494	Cases *n* 138	Controls, *n* 356	*P*-value
Deficiency (≤20 ng/ml)	16⋅60 (13⋅42–20⋅18)	16⋅66 (10⋅86–23⋅95)	16⋅57 (12⋅86–20⋅85)	0⋅980
Insufficiency (21–29 ng/ml)	48⋅58 (44⋅09–53⋅09)	57⋅24 (48⋅54–65⋅62)	45⋅22 (39⋅97–50⋅55)	0⋅016
Sufficiency (≥30 ng/ml)	34⋅82 (30⋅62–39⋅20)	26⋅08 (18⋅98–34⋅24)	38⋅20 (33⋅13–43⋅47)	0⋅011

Prevalence and confidence interval of 95 %.

Table 3 presents the multiple logistic regression analysis, which showed that the insufficiency of vitamin D status was associated with overweight or obesity. In children and adolescents with insufficiency of vitamin D status, overweight or obesity was 1⋅7 times more frequent (OR 1⋅73; 95 % CI 1⋅05–2⋅84; *P*=0⋅031) compared to those with sufficient 25-OH-D levels, even after adjustment diet rich in 25-OH-D, vigorous physical activity, television viewing time and computer use time. The model presented adequate goodness of fit (*P*=0⋅452).

**Table 3.** Multivariable analyses of the association of vitamin D status with overweight or obesity in children and adolescents from Bucaramanga, Colombia (*n* 494)

Overweight or obesity	Model^a^		
	OR	IC 95 %	*P*-value
Sufficiency (≥30 ng/ml)	Reference		
Insufficiency (21–29 ng/ml)	1⋅73	1⋅05–2⋅84	0⋅031
Deficiency (≤20 ng/ml)	1⋅19	0⋅59–2⋅38	0 612

a Model adjusted by the diet rich in 25-OH-D, vigorous physical activity, television viewing time and computer use time.

## Discussion

Our study demonstrated a high prevalence of vitamin D deficiency and insufficiency in this population, with 25-OH-D insufficiency being greater and sufficiency lower in overweight children and adolescents compared to those with normal body weight. Furthermore, 25-OH-D insufficiency was associated with overweight or obesity after adjusting for the main confounding variables.

The high prevalence of vitamin D deficiency and insufficiency in this study could be explained by the inadequate intake of foods rich in vitamin D, such as dairy products, vegetables, animal protein and seafood(^11^). Also, the study population had a low-medium socioeconomic level, a risk factor that has been associated with behaviours that can affect vitamin D status(^17^). The results found by Beer *et al*. mention that indicators of socioeconomic status, including education and wealth, were positively associated with vitamin D deficiency(^18^).

Until now, the nature of the association between low vitamin D status and obesity remains debatable. The exact mechanisms responsible for this association remain unclear, and the clinical utility of increasing vitamin D status to reduce adiposity still requires further evaluation(^19^). Several possible biological mechanisms may explain the relationship between the serum levels of vitamin D and body fat indices, even in a bidirectional fashion. On the other hand, there are also mechanisms in the pathway that explain obesity as a causal factor for low vitamin D levels, such as decreased exposure to the sun(^20^). However, Bucaramanga is located at 7° 07′07 ″ N and 73° 06′58 ″ W; its climate is tropical and isothermal with a generally dry and sunny season from December to March. Therefore, it is not affected by the climatic seasons. Other mechanisms of association between vitamin D and overweight or obesity are widely described in Vranić *et al*., according to the consequence and the cause(^21^). Among them, ‘*its deficiency has been associated with a large number of disorders such as metabolic syndrome, cancers, and autoimmune, psychiatric, and neurodegenerative diseases, but the causative role of VD deficiency in many of these conditions remains unclear. Since VD deficiency is related to visceral adiposity; it could be used as a biomarker of a visceral adiposity-related dysmetabolic state (cardiovascular diseases, type 2 diabetes, dyslipidemia, arterial hypertension)*’(^21^).

The findings of the present investigation are consistent with a study carried out in Bogotá, Colombia. In this, Gilbert-Diamond *et al.*(^12^) evaluated the association between the vitamin D status and the changes in indicators of adiposity and growth in a representative prospective cohort of 479 school-age children (8⋅9 ± 1⋅6 years; 52 % girls) of low and middle income. These children were followed for an average of 29 ± 5⋅1 months, determining an overweight incidence of 11 % and finding an inverse association between vitamin D status and a change in BMI during follow-up. Compared with children with sufficient vitamin D, the change in BMI was 0⋅1 kg/m^2^ per year higher in children with insufficiency and deficiency. A sub-analysis of the previous study(^22^) evaluated the association between vitamin D status with the age of menarche in 242 girls and reported that the prevalence of overweight/obesity was higher (21⋅4 %) in girls with 25-OH-D deficiency status compared to insufficiency states (12⋅3 %) and those with sufficient 25-OHD-D levels (5⋅6 %).

The present results are also consistent with the few investigations carried out in Latin America. A study carried out in Mexican boys and girls between the ages of 6 and 12 years old (*n* 99 obese and *n* 99 non-obese) randomly selected from a population of 954 children from 6 urban public schools, determined an obesity prevalence of 28 %, of which 62⋅1 % had 25-OH-D insufficiency and 20⋅2 % deficiency. No statistically significant differences were found regarding vitamin D insufficiency between obese and non-obese children (64⋅7 *v*. 59⋅6 %; *P*=0⋅549), but deficiency status was significantly different (27⋅3 *v*. 13⋅1 %; *P*=0⋅021). Likewise, in a bivariate analysis, 25-OH-D concentrations were inversely correlated with the percentage of body fat, BMI, triceps skinfold and waist circumference(^23^). Likewise, a Brazilian study investigated the association between the status of 25-OH-D and cardiovascular risk factors in 220 school adolescents (15–19 years old), showing that 57⋅3 and 78 % had vitamin D insufficiency/deficiency and overweight or obesity, respectively. Additionally, vitamin D status was associated with indicators related to overweight (BMI and waist circumference)(^24^). Another more representative study conducted in 1152 adolescents (12–17 years old) from four cities in Brazil established a prevalence of 25-OH-D deficiency of 21 %, insufficiency of 42 % and sufficiency of 37 %(^25^).

Similarly, in northern and southern Europe, Australia and New Zealand, Saudi Arabia, and in white, Afro-descendant, and Hispanic groups in the United States, obese adults and children have been shown to have lower levels of vitamin D in serum when compared to people with normal body weight. In contrast, 25-OH-D levels were inversely correlated with body weight, BMI and body fat(^19^).

One of the strengths of this study was the quantification of their plasma 25-OH-D levels was determined directly and not by indirect estimations such as frequency questionnaires of food consumption that can promote an information bias. However, the results of this study should be interpreted carefully, considering certain methodological limitations. The primary restrain of this study is related to the type of design since there is no possibility of having evidence about the timing and direction of the association between serum vitamin D levels and overweight or obesity. Likewise, the risk of residual confounding is a limitation of their study. Correspondingly, no information was collected on other potential determinants of 25-OH-D levels, such as time spent outdoors, use of sunscreen, measurement of parathyroid hormone levels and calcium intake. Another limitation was the non-quantification of the time of exposure to the sun.

## Conclusion

25-OH-D insufficiency is common in children and adolescents in Bucaramanga, Colombia, and it was associated with overweight or obesity. Clinical trials are necessary to determine the causal relationship between vitamin D deficiency/insufficiency and overweight or obesity. If the evidence from interventional studies is robust, tighter control over the 25-OH-D levels of the population would be needed. Also requiring people to be encouraged to acquire a balanced diet rich in vitamin D and have a longer exposure time to sunlight in order to provide help in the effort of controlling the obesity epidemic worldwide.
